# Risk Factors of Subclinical Atherosclerosis and Plaque Burden in High Risk Individuals: Results From a Community-Based Study

**DOI:** 10.3389/fphys.2018.00739

**Published:** 2018-06-22

**Authors:** Lianduo Bian, Lili Xia, Yixin Wang, Jiajia Jiang, Yonghui Zhang, Dongxue Li, Wei Li, Yan He

**Affiliations:** ^1^Department of General Medicine, Beijing Anzhen Hospital, Capital Medical University, Beijing, China; ^2^Editorial Office of Hepatobiliary and Pancreatic Diseases International, The First Affiliated Hospital, College of Medicine, Zhejiang University, Hangzhou, China; ^3^Department of Epidemiology and Biostatics, School of Public Health, Capital Medical University, Beijing, China

**Keywords:** carotid intima-media thickness, carotid plaque, plaque burden, community-based study, subclinical atherosclerosis

## Abstract

China is going through major change and the incidence of first-ever stroke has increased dramatically. In this study, we aim to determine the ultrasound characteristics of carotid intima-media thickness (CIMT) and carotid plaques (CP) in the Chinese community-based population with high risk of stroke. 1009 stroke-free participants from Datun community were classified at high risk of stroke and included in this cross-sectional study. We performed B-mode carotid ultrasound imaging in all of the study subjects to measure the CIMT in the common carotid artery (CCA) far wall and CP in the CCA, bifurcation and internal carotid artery. Stepwise logistic regression analyses were used to determine factors associated with elevated CIMT and subclinical atherosclerosis, as well as plaque burden (≥2 plaques). Our results showed that traditional risk factors including aging, hypertension, current smoking and the level of high density lipoprotein cholesterol are associated with subclinical atherosclerosis and plaque burden in high-risk community residents. To improve primary prevention in this population, we may consider intense blood pressure and lipid management, and smoking cessation.

## Introduction

Stroke has become a threatening public health issue in China. Recently, a dramatically increased incidence of first-ever stroke among a low-income population in rural China from 1992 to 2012 has been reported, with an annual increase of 6.5% (Wang et al., 2015). Moreover, the prevalence of traditional risk factors for stroke like obesity and hypertension is also booming in this population over the past several decades, especially in those aged 35–44 years (Ning et al., 2014; Wang et al., 2014), indicating an even heavier disease burden in the near future.

B-mode ultrasound is one of the imaging techniques with the merits of non-invasive, low cost and no contradictions, and offers valuable information such as carotid intima thickness (CIMT) and the presence of plaque. These parameters have been reported to predict cardiovascular disease (CVD) in several large studies (Lorenz et al., 2010; Polak et al., 2011). In 2007, a systematic review and meta-analysis conducted by Lorenz et al showed that with every 0.1 mm increase in CIMT, the future risk of myocardial infarction (MI) and stroke increased 10–15 and 13–18%, respectively (Lorenz et al., 2007). The ability of CIMT to predict future CVD was also supported by a number studies conducted in subjects with Asian ancestry (Chuang et al., 2011; Lee et al., 2014). However, the result from Tromso Study showed that the total carotid plaque area was associated with MI and stroke, while CIMT did not predict stroke and the prediction of CIMT for MI no longer existed after excluding carotid bulb IMT (Johnsen et al., 2007; Mathiesen et al., 2011). A meta-analysis that analyzed results from 14 population-based studies and excluded those with plaques to evaluate common carotid artery IMT (CCA-IMT) also demonstrated that the addition of CCA-IMT to the Framingham Risk Score does improve the prediction of first-time MI or stroke a little bit, but the improvement add no clinically meaningful information (Den Ruijter et al., 2012). So the utility of CIMT in the prediction of CVD is quite controversy, and since plaque was predominantly formed in carotid bulb and internal carotid artery, CIMT measured in these sites may include plaque already. Carotid plaque, on the other hand, is more strongly associated with traditional risk factors such as age, sex, hypertension, diabetes and hypercholesterolemia (Moskau et al., 2005) than CIMT. Plaques also correlate with other measures of atherosclerotic vascular disease, such as aortic stiffness (Zureik et al., 2002). Furthermore, echogenic plaques are associated with increased arterial stiffness (Zureik et al., 2003). The results of Three-City Study showed that plaque but not CIMT were independent predictor of CVD and significantly added to CVD risk prediction over conventional risk factors (Plichart et al., 2011).

Considering the rapid growing incidence of stroke in China and its severe consequences, studies of risk factors associated with subclinical atherosclerosis and plaque burden are warranted, especially for those at high risk of stroke. The aim of this community-based population study was to determine the risk factors for CIMT and carotid plaques (CP) in the subjects at high risk of stroke. This study will provide the rationale to propose intervention and management in this population.

## Materials and Methods

### Study Population and Design

The data were collected form Datun community, Chaoyang District of Beijing, China in 2012, which was one of the sites of the China National Stroke Prevention Project (CSPP) (Longde et al., 2015). In brief, all residents aged 40 years old or over in the community were invited to complete a screen questionnaire, and from which subjects at high-risk of stroke were identified and recruited, and a further physical examination and laboratory measurements were performed among these participants.

Data on demographic characteristics, lifestyle risk factors and medical history were obtained by face-to-face interviews with a questionnaire. Smoking and drinking status were asked in details according to the answers to “Are you currently a smoker/drinker.” If the answer was yes, then participants were asked how many years have they been smoking/drinking and the amount per day. If participants used to be a smoker/drinker, they were asked how many years have they quitted smoking/drinking. Answers were confirmed by participants’ spouses. Smoking and drinking status were categorized as current and non-current smoker/drinker.

The Ethics Committee of the China CSPP, including key neurologists, cardiologists, and epidemiologists, was established to provide ethnical approval and technical support to the program (No. 023-2011). Written informed consent was obtained from each participant.

### Identified Subjects at High Risk of Stroke

Screen questionnaire included questions as follows:

(1) blood pressure ≥140/90 mmHg, (2) atrial fibrillation or valvular heart disease, (3) smoking, (4) dyslipidemia (triglyceride ≥2.26 mmol/L or total cholesterol ≥6.22 mmol/L or low density lipoprotein cholesterol ≥4.14 or high density lipoprotein cholesterol < 1.04mml/L), (5) diabetes, defined according to 1999 World Health Organization criteria, (6) lack of exercise, defined as less than 3 times per week and less than 30 min each time, (7) BMI ≥ 26, (8) having family history of stroke. Those having 3 or more risk factors listed in the screen questionnaire or with a history of stroke were defined as high-risk individuals. Of over 6000 residents screened, 1197 were found to be at high risk. After excluding those with reported history of stroke (*n* = 159), and those without ultrasound examination (*n* = 28), a total of 1009 stroke-free participants with the age range of 40–84 years old were included in this study.

### Physical Examination and Laboratory Measurements

Height and weight were measured with a standard process using a SECA 813 digital scale (Seca, Vogel & Halke GmbH & Co., Hamburg, Germany) and a flexible anthropometer as we previously reported (Xia et al., 2017). Waist circumference was measured at the midpoint between the lower border of the rib cage and the iliac crest. Body mass index (BMI) was calculated as body weight in kilogram divided by body height squired in meters. Blood pressure (BP) and pulse were measured in triplicate after 10-min rest with Omron electronic sphygmomanometers (Omron Healthcare Co. Ltd., Kyoto Japan).

Venous blood samples were obtained after an overnight fast of at least 8 h. All blood samples were analyzed in a national central laboratory in Beijing using the Olympus auto-analyzer 2700 (Olympus Instruments Inc., Tokyo, Japan), with strict quality control. Fasting glucose, serum triglyceride (TG), low-density lipoprotein cholesterol (LDLC) and high-density lipoprotein cholesterol (HDL-C) concentrations were measured as we previously described (Xia et al., 2017). HbA1c was measured by high-performance liquid chromatography (HPLC) using the Variant II (Bio-Rad, Hercules, CA, United States). Serum levels of homocysteine (HCY) was estimated using commercial kits (Homocysteine Assay Kit [AB200], Ausa Pharmed Co., Ltd., Shenzhen, China).

### Nonmydriatic Direct Ophthalmoscopy

Ophthalmologists performed direct ophthalmoscopy without mydriasis after a general physical examination. According to Scheie’s classification (Scheie, 1953), Fundus arteriosclerosis was defined as the presence of broadening of the light reflex from artery, arterial narrowing, arteriovenous compression, and “copper” or “silver” wiring. Mild fundus arteriosclerosis were defined as stage 1 in Scheie’s classification and moderate to severe fundus arteriosclerosis was defined as stage 2–4 in Scheie’s classification.

### Ultrasound Examination

All subjects were studied lying in supine position with their head turned 45 degrees from the site being scanned, and the operator seated at the head bed, using a Philips HDI 5000 ultrasound system equipped with a 7.5 MHz probe. During ultrasound examination, the common carotid intima-media thickness (CIMT), the presence and type of carotid plaque were recorded. Carotid IMT scanning and reading was performed as suggested by the Mannheim Consensus (Touboul et al., 2004). The IMT was defined as the distance between the leading edge of the lumen-intima echo and the leading edge of the media adventitia echo. The CIMT was defined as the mean of the right and left IMT of the CCA far wall. Elevated CIMT was defined as CIMT thickening over 1mm, as suggested in literature (Howard et al., 1993). Carotid plaques were defined as a focal region with a thickness >1.5 mm as measured from the media adventitia interface to the lumen-intima interface or as the presence of focal wall thickening that is at least 50% greater than that of the surrounding vessel wall. According to the characteristics of CP echogenicity, CP were categorized into three groups, namely, echogenic (higher content of fibrous tissue and calcification), echolucent (lipid rich), heterogeneous (mixed echolucent and echogenic) (European Carotid Plaque Study Group, 1995; Mathiesen et al., 2001). Presence of carotid plaque was defined as subclinical atherosclerosis.

### Statistical Analysis

All statistical analysis were conducted using SPSS 23.0 software (IBM Corp, Chicago, IL, United States). Participants were divided into three groups according to CIMT (CIMT < 1mm; CIMT 1 ∼ 1.5 mm) and subclinical atherosclerosis (presence of carotid plaque). The data was expressed as mean ± standard deviation (SD) for the continuous variables and number (percentage) for the categorical variables, respectively. Analysis of variance (ANOVA) was used to compare the continuous variables among the three groups, and Chi-square test was for the comparison of categorical variables. Stepwise logistic regression analyses were used to evaluate the factors associated with elevated CIMT and subclinical atherosclerosis, and this model was also used to determine factors associated with plaque burden (plaque ≥2). Two-sided *P*-values <0.05 were considered statistically significant.

## Results

### General Characteristics of Study Population

A total of 1009 individuals (305 males and 704 females) at high risk of stroke were included. The mean age of the subjects was 61.7 ± 9.0 years. The proportion of subjects at subclinical atherosclerosis was 81.5% (822), including 147 (14.6%) subjects with CIMT and 675 (66.9%) subjects with carotid plaque/subclinical atherosclerosis. Among those with carotid plaque, the proportion of having echolucent, echogenic and heterogeneous plaque were 17.9, 22.9, and 59.2%, respectively.

**Table 1** showed the clinical characteristics of the subjects. As compared to the reference group (CIMT < 1 mm), subjects with elevated CIMT and subclinical atherosclerosis were older, more likely to be males, current smoker and current drinker, having history of hypertension and diabetes, having moderate-to-severe fundus arteriosclerosis and having less favorable metabolic profiles, such as elevated levels of systolic BP, fasting plasma glucose and homocysteine.

**Table 1:** Characteristics of subjects according to carotid intima-media thickness and subclinical atherosclerosis.

Variables	Total	CIMT < 1 mm	Elevated CIMT (1 mm ≤ CIMT ≤ 1.5 mm)	Subclinical atherosclerosis	*P*
N (%)	1009 (100)	187 (18.5)	147 (14.6)	675 (66.9)	
Age, years	61.7 ± 9.0	56.5 ± 8.4	59.6 ± 8.2	63.7 ± 8.6	< 0.001
Male, n (%)	276 (27.4)	33 (17.6)	29 (19.7)	243 (36.0)	< 0.001
Current smoker, n (%)	161 (16.0)	16 (8.6)	18 (12.2)	127 (18.8)	0.002
Current drinker, n (%)	207 (20.5)	23 (12.3)	32 (21.8)	152 (22.5)	0.008
Hypertension, n (%)	578 (57.3)	86 (46.0)	72 (49.0)	420 (62.2)	< 0.001
Diabetes, n (%)	229 (22.7)	29 (15.5)	24 (16.3)	176 (26.1)	0.001
Dyslipidemia, n (%)	586 (58.1)	104 (55.6)	87 (59.2)	395 (58.1)	0.743
**Plaque composition, n (%)**					
Echolucent	124 (12.3)			124 (17.9)	
Echogenic	158 (15.7)			158 (22.9)	
Heterogeneous	409 (40.5)			409 (59.2)	
**Fundus arteriosclerosis, n (%)**					
normal	450 (44.6)	100 (53.5)	64 (43.5)	286 (42.3)	<0.001
mild	275 (27.2)	62 (33.2)	35 (23.8)	178 (26.4)	
moderate to severe	284 (28.1)	25 (13.3)	48 (32.7)	211 (31.3)	
Lack of exercise, n (%)	474 (47.0)	98 (52.4)	71 (48.3)	305 (45.2)	0.203
Body mass index, mg/m^2^	26.0 ± 3.7	26.0 ± 4.2	26.6 ± 3.9	25.9 ± 3.5	0.087
WC, cm	86.4 ± 9.7	85.2 ± 10.1	86.7 ± 9.8	86.6 ± 9.5	0.199
SBP, mmHg	126.2 ± 14.5	123.5 ± 14.2	125.3 ± 14.4	127.2 ± 14.5	0.005
DBP, mmHg	78.3 ± 7.9	77.6 ± 8.8	78.8 ± 7.5	78.4 ± 7.8	0.350
FPG, mmol/L	5.40 ± 1.49	5.26 ± 1.33	5.20 ± 1.17	5.49 ± 1.59	0.038
HbA1c, %	6.01 ± 0.91	6.12 ± 1.06	6.11 ± 0.96	5.95 ± 0.85	0.053
TG, mmol/L	1.54 (1.10–2.13)	1.56 (2.06–2.34)	1.59 (1.14–2.19)	1.60 (1.16–2.18)	0.983
TC, mmol/L	5.33 ± 1.07	5.25 ± 1.05	5.24 ± 1.05	5.35 ± 1.08	0.627
HDL-C, mmol/L	1.30 ± 0.30	1.34 ± 0.30	1.28 ± 0.27	1.28 ± 0.30	0.075
LDL-C, mmol/L	3.07 ± 0.86	2.98 ± 0.83	3.11 ± 0.84	3.08 ± 0.87	0.311
HCY, umol/L	17.31 ± 5.97	16.68 ± 5.44	16.25 ± 3.84	17.73 ± 6.40	0.006

*Data are expressed as mean ± SD or number (percentage). SBP, systolic blood pressure; DBP, diastolic blood pressure; FPG, fasting plasma glucose; TC, total cholesterol; TG, triglyceride; HDL-C, high density lipoprotein cholesterol; LDL-C, low density lipoprotein cholesterol; HCY, homocysteine; WC, waist circumference. P-values were from ANOVA or Chi-square test.*

### Factors Associated With Elevated CIMT and Subclinical Atherosclerosis

Stepwise logistic regression revealed that current drinker [OR and 95% CI: 3.44 (1.55–7.63), *P* = 0.002] and moderate-to-severe fundus arteriosclerosis [OR and 95% CI: 3.40 (1.67–6.91), *P* = 0.001] increased the risk of elevated CIMT, while each 1 mmol/L increase of HDL-C decreased 67% risk. Compared to those with normal CIMT (CIMT < 1mm), for subclinical atherosclerosis, age and current smoker were significantly associated with increased odds and HDL-C was protective factors (OR: 0.40 and 0.51, 95% CI: 0.21–0.75 and 0.29–0.90, respectively). Moreover, moderate-to-severe fundus arteriosclerosis increased 75% risk of having subclinical atherosclerosis. Compared to those with elevated CIMT (1 ≤ CIMT ≤ 1.5 mm), aging was associated with increased risk and females were at lower risk of subclinical atherosclerosis than males, when elevated FPG [OR = 1.31 (1.05–1.65)] and history of hypertension [OR = 1.69 (1.08–2.66)] were risk factors. (**Table 2**).

**Table 2:** Factors associated with elevated CMIT and subclinical atherosclerosis.

Variables	1 ≤ CIMT ≤ 1.5 mm (*n* = 147) vs. CIMT < 1mm		Subclinical atherosclerosis (*n* = 675) vs. CIMT < 1mm		Subclinical atherosclerosis (*n* = 675) vs. 1 ≤ CIMT ≤ 1.5 mm	
			
	OR (95% CI)	*P*-value	OR (95% CI)	*P*-value	OR (95% CI)	*P*-value
Age, years			1.11 (1.08–1.13)	<0.001	1.04 (1.02–1.07)	0.002
HDL-C, mmol/L	0.33 (0.12–0.88)	0.027	0.40 (0.21–0.75)	0.004		
Female gender			0.55 (0.33–0.91)	0.019	0.44 (0.25–0.77)	0.004
Current smoker			2.65 (1.35–5.20)	0.005		
Current drinker	3.44 (1.55–7.63)	0.002				
normal	reference					
mild	0.90 (0.49–1.66)	0.742				
moderate to severe	3.40 (1.67–6.91)	0.001				
FPG					1.31 (1.05–1.65)	0.014
Hypertension					1.69 (1.08–2.66)	0.022

*Variables that resulted statistically significant at p ≤ 0.10 in the univariate analysis, which included sex, age, BMI, SBP, FPG, HbA1C, HDLC, HCY, hypertension, diabetes, fundus arteriosclerosis, current smoking and drinking status, were introduced in a multivariate logistic regression model and a backward selection method was used with a significance level of 0.05.*

### Factors Associated With Carotid Plaque Burden (Plaques ≥2)

We further investigated the factors associated with carotid plaque burden (plaques ≥2) using stepwise logistic regression analysis (**Table 3**). The results showed that sex, age, hypertension, diabetes, current smoker, waist circumference, HDL-C and LDL-C were independently associated with carotid plaque burden, with female gender [OR and 95% CI: 0.40 (0.26–0.63), *P* < 0.001], waist circumference [OR and 95% CI: 0.98 (0.96–1.00), *P* = 0.012] and HDL-C [OR and 95% CI: 0.39 (0.20–0.76), *P* = 0.005] considered protective factors, and age [OR and 95% CI: 1.11 (1.08–1.13), *P* < 0.001], hypertension [OR and 95% CI: 1.77 (1.25–2.50), *P* < 0.001], diabetes [OR and 95% CI: 1.68 (1.13–2.50), *P* = 0.010], current smoking [OR and 95% CI: 2.05 (1.19–3.54), *P* = 0.010] and LDL-C [OR and 95% CI: 1.24 (1.02–1.51), *P* = 0.029] considered risk factors. In those individuals with carotid plaque, age and FPG increased the odds of having ≥2 plaques (OR value were 1.06 and 1.14, respectively) while HDL-C and female gender decreased the odds (OR value were 0.43 and 0.41, respectively).

**Table 3:** Stepwise logistic regression for carotid plaque burden (≥2 plaques).

Variables	no plaque (*n* = 304)	1 plaque (*n* = 228)	≥ 2 plaques (*n* = 447)	≥2 plaques vs. 1 plaque		≥2 plaques vs. no plaque	
			
	$\bar{X}$ ± SD/n (%)	$\bar{X}$ ± SD/n (%)	$\bar{X}$ ± SD/n (%)	OR (95% CI)	*P*-value	OR (95% CI)	*P*-value
Age, years	57.9 ± 8.5	61.4 ± 8.4	64.9 ± 8.4	1.06 (1.03–1.08)	<0.001	1.11 (1.08–1.13)	<0.001
WC, cm	85.9 ± 10.0	85.2 ± 9.9	87.4 ± 9.3			0.98 (0.96–1.00)	0.012
HDL-C, mmol/L	1.32 ± 0.29	1.35 ± 0.32	1.25 ± 0.29	0.43 (0.24–0.77)	0.005	0.39 (0.20–0.76)	0.005
FPG, mmol/L	5.24 ± 1.26	5.30 ± 1.15	5.58 ± 1.77	1.14 (1.00–1.29)	0.043		
Female gender	249 (81.9)	178 (78.1)	254 (56.8)	0.41 (0.28–0.60)	<0.001	0.40 (0.26–0.63)	<0.001
Current smoker	32 (10.5)	27 (11.8)	99 (22.1)			2.05 (1.19–3.54)	0.010
Hypertension	137 (45.1)	129 (56.6)	291 (65.1)			1.77 (1.25–2.50)	0.001

*Variables that resulted statistically significant at p ≤ 0.10 in the univariate analysis, which included sex, age, WC, SBP, FPG, HDLC, hypertension, diabetes, fundus arteriosclerosis, current smoking and drinking status, were introduced in a multivariate logistic regression model and a backward selection method was used with a significance level of 0.05.*

## Discussion

We sought to investigate the determinants of subclinical atherosclerosis and plaque burden in a community-based, cross-sectional study of Chinese stroke-free but in asymptomatic high-risk population, of which about 15% had elevated CIMT and 67% had carotid plaque/subclinical atherosclerosis. Our study demonstrated that among these high risk individuals, aging, HDL-C, current smoking, current drinking and moderate-to-severe fundus arteriosclerosis were determinants of subclinical atherosclerosis. Furthermore, we found that aging, current smoking and hypertension were associated with increased risk of plaque burden (plaque ≥2), and that females were at decreased risk. In particular, female gender and increase in HDL-C decreased the risk of having multiple plaques, while aging and increase in FPG increased the risk in those with carotid plaque.

Carotid intima media is composed of smooth muscle cells (media layer, 80%) and endothelium (intima, 20%), which cannot be discriminated by current ultrasound technique. Besides intimal thickening, increase in this combined thickness (namely, CIMT) represents smooth muscle hypertrophy and/or hyperplasia, which may be induced by pressure overload and/or age-related sclerosis (Finn et al., 2010). Our study found that current drinking and moderate-to-severe fundus arteriosclerosis was associated with elevated CIMT. The association between fundus abnormality and elevated CIMT found in our study has been consistently reported in other studies. In a Chinese community-based study of diabetes patients aged 40 years or older, the presence of diabetic retinopathy was significantly associated with increased odds of subclinical atherosclerosis (Liu et al., 2015). Moreover, CIMT was negatively associated with retinal arteriolar caliber and positively associated with retinal venular caliber in hypertension patients, demonstrating that vascular damage occurs in parallel in large arteries and in the microcirculation (Torres et al., 2013). Mechanism of these associations are largely unknown, but shared pathophysiological mechanisms beyond traditional cardiovascular risk factors have been suggested (Alonso et al., 2015).

Plaque, on the other hand, is a localized manifestation of atherosclerosis, which represents predominantly intimal thickening and is more likely related to inflammation, oxidation, endothelial dysfunction, and/or smooth cell proliferation (Mathiesen and Johnsen, 2009). In our study, carotid plaque was associated with aging, smoking and level of HDL-C, and carotid plaque burden (≥2 plaques) was associated with aging, smoking, gender, hypertension, and levels of HDL-C and FPG. In consistency with our study, a previous study has shown that factors independently associated with the presence of carotid plaque were age, past smoke, HDL cholesterol, retinopathy and retinopathy plus nephropathy in diabetes patients aged over 45 years (de Kreutzenberg et al., 2011). David Spence et al also reported that traditional risk factors including age, sex, hyperlipidemia, hypertension and smoking were determinants of carotid plaque area, which accounted for about 60% of the variation in carotid plaque area (Spence et al., 1999). Moreover, Núria Alonso et al recently demonstrated that aging, gender, dyslipidaemia and the presence of retinopathy were associated with the presence of ≥2 CP in diabetes patients (Alonso et al., 2015).

Unlike CIMT which has an undetectable change with the annual change roughly 0.01–0.04 mm, CP grow 2.4 times faster in longitudinal view, which allows assessments of the progression of disease in months (Spence and Hackam, 2010). The Tromso Study measured IMT and total plaque area in 3240 men and 3344 women with the age range of 25–84 and followed these participants 10 years. They found that every 1 SD in square-root-transformed plaque area increased the risk of incident ischemic strokes by 9–38% in man and 1–41% in women. Furthermore, the multivariable-adjusted model showed that compared to no plaque, the highest quartile of plaque area increased 0.73- and 0.62-folds the risk of stroke in man and women, respectively (Mathiesen et al., 2011). On the other hand, the results of Three-City Study showed that plaques were independent predictors of CVD risk and significantly added to CVD risk prediction over conventional risk factors. The study also showed that hazard ratio of incident CVD increased as the number of plaque increased, with plaque at one site predicted 1.5-folds the risk when plaque at two or more sites predicted 1.5-folds the risk (Plichart et al., 2011). Moreover, the Northern Manhattan Study showed that plaque thicken over 1.9 mm was associated with 2.8-fold increased risk of vascular events (Rundek et al., 2008). A recent study also showed that the presence of CP, history of smoking, and statin therapy might be important factors for primary prevention of first atherosclerotic CVD in asymptomatic high-risk patients, especially in middle-aged patients, emphasizing the importance of carotid artery evaluation (Kim et al., 2015).

Smoking was a common risk factor for subclinical atherosclerosis and plaque burden in our study, rising about 1-fold risk than those non-smokers, which was similar to previous studies (McEvoy et al., 2015; Hisamatsu et al., 2016) The evidence linking smoking exposure with various CVD including MI, stroke and peripheral vascular diseases, is clearly present (Ambrose and Barua, 2004). Atherosclerosis is considered as a critical player in the pathophysiology of smoking-induced CVD, and these adverse effects would be reduced by smoking cessation (Johnson et al., 2010). Supporting this notion, recently, the Multi-Ethnic Study of Atherosclerosis (MESA) and the Shiga Epidemiological Study of Subclinical Atherosclerosis (SESSA), both of which were community-based cohort, have reported the association of current and former smoking to CAC (coronary artery calcium), CIMT and ABI (ankle-brachial index)—subclinical atherosclerosis at 3 anatomically distinct sites— in the western and Japan population, respectively (McEvoy et al., 2015; Hisamatsu et al., 2016). A monotonic relationship was also found between increasing pack-years exposure and elevated inflammatory markers and time since quitting in former-smokers was independently associated with lower inflammation and atherosclerosis in MESA (McEvoy et al., 2015). Similarly, in SESSA, researchers observed dose–response relationships of pack-years and daily consumption, with CIMT, carotid plaque, AoAC (aortic artery calcification), and ABI among both current and former smokers, and even a small amount of pack-years or daily consumption among current smokers was associated with coronary artery calcification and AoAC, whereas time since cessation among former smokers was linearly associated with lower burdens of all atherosclerotic indices (Hisamatsu et al., 2016).

There were some limitation to this study. First, this was a cross-sectional study and was not randomized. Additionally, we did not compare the characteristics of those excluded in the screening process (identified as non-high risk) with those included (identified as high risk) in our study due to lack of data, so our results can only apply to those at high risk of stroke. Second, we only measured the CCA far wall IMT, and used arbitrary cutoff value to define elevated CIMT. However, the cutoff value is widely used, and CCA far wall IMT is validated as representing the true thickness of the vessel wall and its measurement is easier and more reliable than the other three segment. Our study also have some strength. First, we first investigated the determinants of subclinical atherosclerosis and plaque burden in a community-based, Chinese stroke-free but at asymptomatic high-risk population. With the booming incidence of stroke in China, such study is in urgent demand. Second, our result may offer some information for community-based interventions for stroke, which is already a common strategy for chronic diseases in China.

## Conclusion

In conclusion, our study showed that traditional risk factors including aging, hypertension, current smoking and the level of HDL-C are associated with subclinical atherosclerosis and plaque burden in high-risk community residents. To improve primary prevention in this population, we may consider intense BP and lipid management, and more importantly, smoking cessation.

## Author Contributions

YH and LB conceived and designed the study. LB, YW, JJ, YZ, DL, and WL acquired the data. LX analyzed and interpreted the data. LX drafted the manuscript. All authors approved the final version to be submitted for publication.

## Conflict of Interest Statement

The authors declare that the research was conducted in the absence of any commercial or financial relationships that could be construed as a potential conflict of interest.
